# Supplementary material for: Prognostic Value of a New Right Ventricular-to-Pulmonary Artery Coupling Parameter Using Right Ventricular Longitudinal Shortening Fraction in Patients Undergoing Transcatheter Aortic Valve Replacement: A Prospective Echocardiography Study
Source: J Clin Med. 2024 Feb 9;13(4):1006. doi: 10.3390/jcm13041006 (PMC10889072; doi:10.3390/jcm13041006)
Supplement: Supplementary file 1 [file jcm-13-01006-s001.zip › jcm-2806355-supplementary.pdf]

Supplementary Files

The TAVR procedure at Amiens-Picardie Hospital

Each patient undergoing TAVR received a comprehensive pre-TAVR assessment performed in the months leading up to the procedure. This assessment included the following supplementary examinations: an electrocardiogram, transthoracic echocardiography, coronary angiography with angioplasty if necessary, an aortic angiographic scan, geriatric assessment, and, for select patients, pulmonary function tests. Each case was subsequently deliberated in a Multidisciplinary Heart Team meeting.

Patients were scheduled for a preoperative appointment on the day before the intervention, during which they underwent a repeat echocardiographic evaluation and a comprehensive blood analysis. The procedure occurred in a hybrid operating room with two interventional cardiologists and an anesthesiologist. A cardiac and vascular surgeon was on standby within the hospital if surgical conversion became necessary.

After the TAVR procedure, patients were monitored for at least 24 hours in the cardiovascular intensive care unit under continuous telemetry surveillance before being transferred to the regular cardiology ward. A follow-up echocardiogram was performed within 72 hours post-procedure to ensure the absence of complications, including paravalvular leaks, prosthesis malposition, and assessment of Vmax and mean gradient."

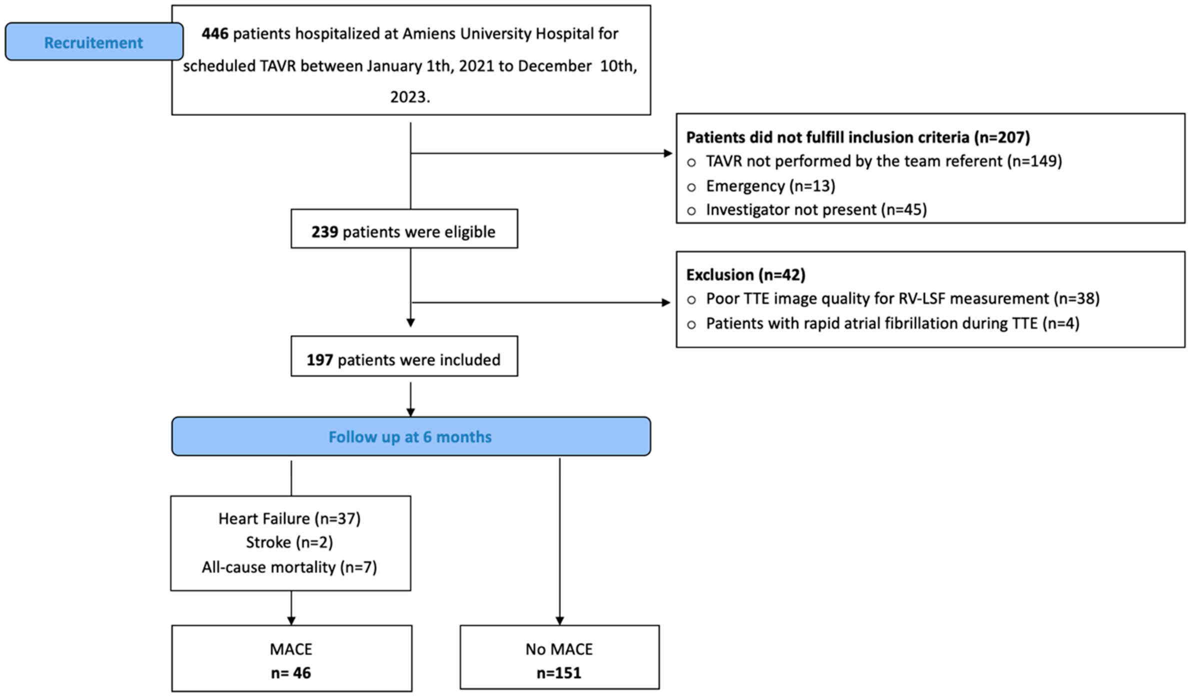

Figure S1 Supplementary Files: Flow chart of the study.

MACE: major cardiovascular clinical event; RV: right ventricular; TAVR: transcatheter aortic valvular replacement; TTE: transthoracic echocardiography

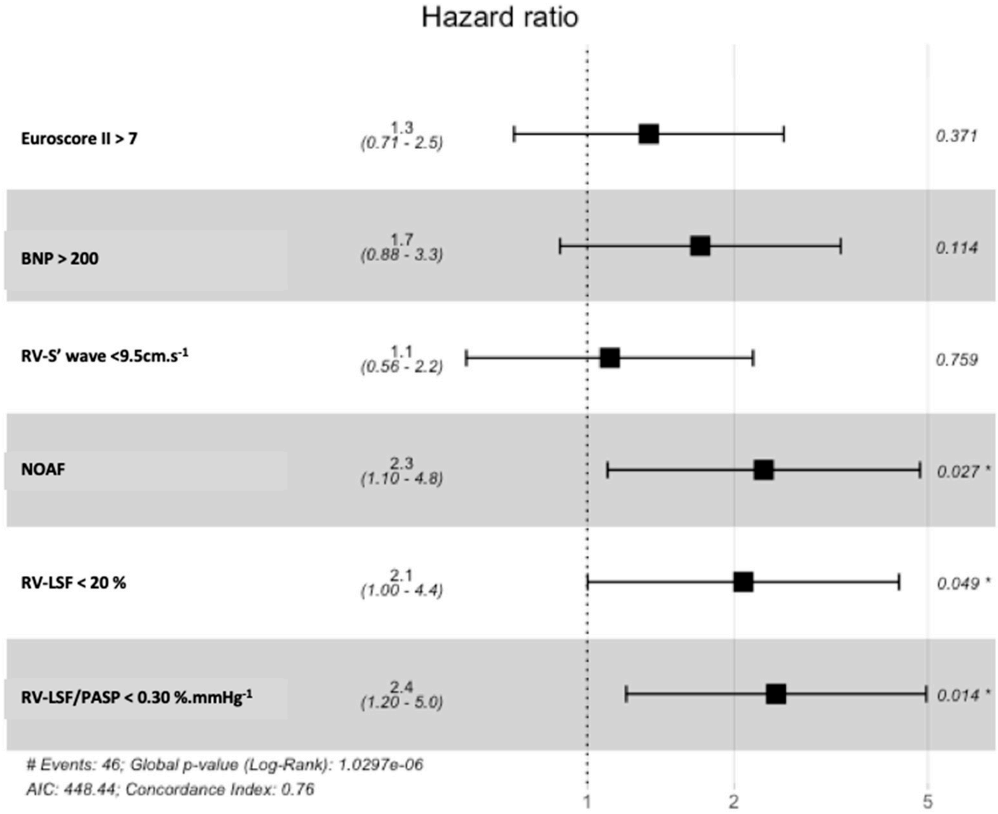

Figure S2 Supplementary Files: Model C of the multivariable analysis.

NOAF: new-onset atrial fibrillation; **PASP**: pulmonary arterial systolic pressure; **RV**: right ventricular; **RV-LSF**: right ventricle longitudinal shortening fraction

Table S1: Demographic data of the general population.

Variables	General Population N=197
Age, years	81 [76-85]
BMI, kg.m^{-2}	27.3 [24.2-30.1]
Female gender, n (%)	80 (41)
Euroscore II, (%)	5.51 [3.69-9.07]
Charlson Score	6.0 [5.0-7.0]
Hypertension	166 (84)
Diabetes mellitus	57 (29)
Dyslipidemia	95 (48)
Smoking	60 (30)
Obstructive sleep apnoea	19 (10)
Peripheareal artery disease	19 (10)
Chronic renal disease	44 (22)
Cardiac surgery	15 (8)
Chronic coronary disease	42 (21)
Myocardial infarction	19 (10)
Atrial fibrillation	63 (32)
COPD	29 (15)
NYHA	2 [2-3]
Biology before TAVR	
BNP level, ng/l	205 [125-449]
Calcic score	2869 [2038-3984]
Hemoglobin, g/dl	12.6 [11.7-13.7]
Creatinin, µmol/l	93.0 [72.0-115.0]

Data are expressed as median (25-50) and count (%).

BMI : body mass index; **COPD** : Chronic obstructive pulmonary disease; **NYHA** : New York Heart Association; **BNP** : brain natriuretic peptide.
